# Why Empirical Forgetting Curves Deviate from Actual Forgetting Rates: A Distribution Model of Forgetting

**DOI:** 10.3390/bs15070924

**Published:** 2025-07-09

**Authors:** Nate Kornell, Robert A. Bjork

**Affiliations:** 1 Department of Psychology, Williams College, 18 Hoxsey Street, Williamstown, MA 01267, USA; 2 Department of Psychology, University of California, Los Angeles, CA 90095, USA; rabjork@psych.ucla.edu

**Keywords:** forgetting, memory, distribution, threshold, learning

## Abstract

For over a century, forgetting research has shown that recall decreases along a power or exponential function over time. It is tempting to assume that empirical forgetting curves are equivalent to the rate at which individual memories are forgotten. This assumption would be erroneous, because forgetting curves are influenced by an often-neglected factor: the distribution of memory strengths relative to a recall threshold. For example, if memories with normally distributed initial strengths were forgotten at a linear rate, percent correct would not be linear, it would decrease rapidly when the peak of the distribution was crossing the recall threshold and slowly when one of the tails was crossing the threshold. We describe a distribution model of memory that explains the divergence between forgetting curves and item forgetting rates. The model predicts that forgetting curves can be approximately linear (or even concave, like the right side of a frown) when percent correct is high. This prediction was supported by previous evidence and an experiment where participants learned word pairs to a criterion. Beyond its theoretical implications, the distribution model also has implications for education: Creating memories that are just above the threshold helps on short-term tests but does not form lasting memories.

## 1. Introduction

Previous research has investigated the mathematical function that describes the rate at which memories are forgotten over time (i.e., the forgetting function), dating back to Ebbinghaus (1885). Classic papers have presented empirical data and posited a variety of theories and mathematical functions to describe shapes of forgetting curves (R. B. Anderson, 2001; R. B. Anderson & Tweney, 1997; Averell & Heathcote, 2011; Donkin & Nosofsky, 2012; Loftus, 1985; Wickens, 1998; Wixted, 2004; Wixted & Ebbesen, 1991).

**Figure 1:**
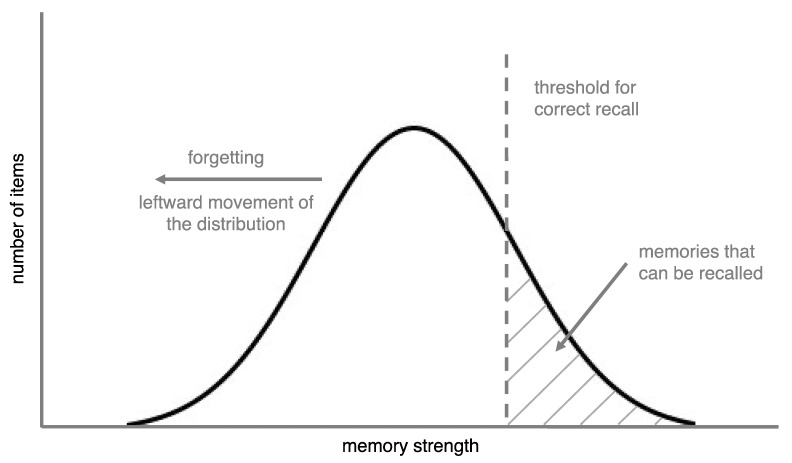
A depiction of forgetting in the distribution model. In this case, a set of memory strengths is normally distributed. Forgetting is represented by the items moving leftward (i.e., losing strength). The percentage of items that are recalled correctly (i.e., that have strengths above the recall threshold) changes as a function of both the rate at which the items move leftward and the shape of the distribution.

In this article, we take a different approach to understand forgetting curves by emphasizing a factor that is often overlooked in forgetting research: the distribution of memory strengths of the items that are being forgotten (see Figure 1). To do this, we define forgetting in two ways: At a theoretical level, forgetting is a decrease in memory strength, while at the level of empirical observation, it is a change whereby a memory that was previously retrievable can no longer be retrieved. For now, we will define memory strength as how accessible a memory is (we provide a more detailed definition later).

Kornell et al. (2011) demonstrated that memory strength distributions can impact empirically observed forgetting rates (this study is described later). Building on this idea, we propose a simple distribution model of forgetting, which posits that these distributions can affect the shape of an empirically observed forgetting curve. Following this simple proposal, we outline predictions of the model, present supporting data, and then sketch outlines of more complicated versions of the model.

The distribution model is not a standalone theory of forgetting, nor is it meant to contradict or replace existing theories. Theories of forgetting such as interference theory (M. Anderson, 2003; Mensink & Raaijmakers, 1988; Underwood, 1957), decay theory (J. Brown, 1958; Hardt et al., 2013), and others (see Oberauer & Lewandowsky, 2008; Wixted, 2004) examine the causes of forgetting at the level of individual items. The distribution model does not focus on individual items but instead takes as given that items are forgotten and adds a second factor, the effect of distributions, to explain empirical forgetting curves. Thus, the distribution model is consistent with existing theories and is an idea that could be added to future theories of forgetting. For these reasons, this article does not provide a comprehensive literature review. The goal of this article is to offer what journalists call an “explainer” (Bahr, 2023): a clear and concise explanation of the distribution model and its relationship to forgetting.

## 2. An Overview of the Distribution Model

The distribution model makes two key assumptions. The first, which seems self-evident, is that different memories have different strengths. The second is that items are recalled if their memory strength is above a threshold. Thresholds are intrinsic to empirical forgetting curves because the percentage of items recalled is, essentially, the percentage of items that are above the threshold. Thus, both key assumptions of the distribution model seem applicable to research on forgetting curves.

**Figure 2:**
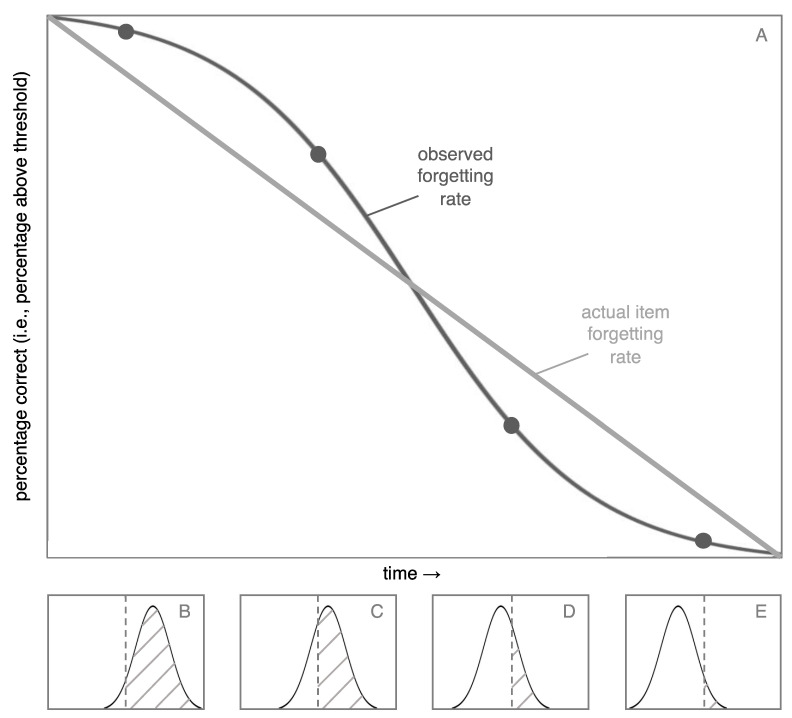
Panel (**A**) depicts the percentage of items above the threshold when a normal distribution moves across a threshold at a constant rate (i.e., when items are forgotten at a linear rate). Panels (**B**–**E**) depict four histograms, with the percentage of items above the threshold (the dashed vertical line) shaded in. Each histogram corresponds to one of the four time points that are identified with dots in panel (**A**). The histogram moves left at a linear rate. In statistical terminology, the proportion of items that are below the threshold in a histogram is referred to as the cumulative distribution function (panel (**A**)). When the underlying distribution is normal, the resulting cumulative distribution function will be s-shaped, which is called a sigmoid function. Thus, panel (**A**) depicts a sigmoid cumulative distribution function, except that it shows the percentage of items above the threshold, rather than below the threshold.

For an illustration of the distribution model, imagine that you studied 100 word pairs and that each pair had a unique amount of strength in your memory. Further, imagine that these memory strengths fit a normal distribution. Assume that all of these items lost strength (i.e., were forgotten) at the same rate and, to keep things simple (and unrealistic, for now), that the rate of forgetting was linear. Finally, assume that memories are only recalled if they are above a memory strength threshold. Figure 2 shows the percentage of items you would recall in this scenario (i.e., percentage of items above the threshold) as a function of time.

The item forgetting rate is linear, so why is the observed forgetting rate non-linear? The answer lies in the four histograms at the bottom of Figure 2. At first (panel B), relatively few items cross the threshold, because there are few items at the tail of the distribution. The observed forgetting is determined by the number of items that cross the threshold from remembered to forgotten. Thus, there is a relatively low rate of observed forgetting. Then, as the middle of the distribution crosses the threshold, more items are forgotten per unit of time (e.g., between panels C and D). Finally, the observed forgetting returns to a low rate (panel E). The point of Figure 2 is that there are systematic differences between underlying forgetting rates (in this case, linear) and empirically observed forgetting rates (in this case, non-linear).

**Figure 3:**
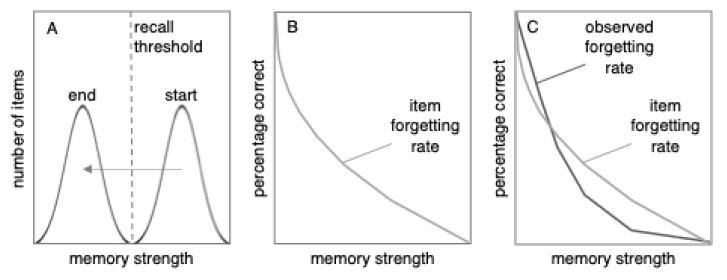
According to the distribution model, an empirically observed forgetting curve (panel (**C**)) is influenced by the distribution of memory strengths (panel (**A**)) and the rate at which individual items are forgotten (panel (**B**)). The item forgetting rate modeled in panels (**B**,**C**) is a power function. The observed forgetting rate in panel (**C**) was created using this function as the item forgetting rate in a simulation of the distribution model.

What happens when we discard the unrealistic idea of a linear forgetting function? Figure 3 makes the more realistic assumption that the forgetting rate of individual items fits a power function (although we are not advocating that power functions are more or less realistic than exponential functions). As Figure 3 shows, the distribution model predicts that the observed forgetting rate (i.e., percent correct) will differ from the item forgetting rate.

## 3. Predictions of the Distribution Model

The distribution model makes testable predictions. Next, we describe three predictions of the model: The observed forgetting rates should be impacted by (a) the section of the distribution being observed, (b) the shape of the distribution, and (c) the standard deviation of the distribution.

### 3.1. Predictions Based on the Section of the Distribution Being Observed

**Figure 4:**
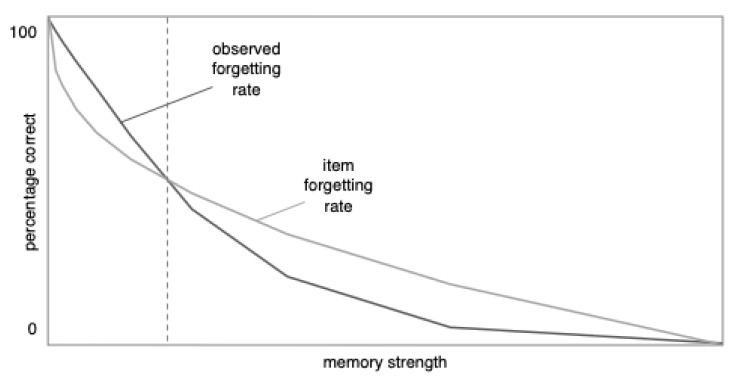
When percent correct is relatively high (to the left of the dashed line), observed forgetting is closer to linear. When percent correct is relatively low, the opposite occurs; observed forgetting is curvier (i.e., has a smaller radius) than the item forgetting curve. This figure was created by enlarging Figure 3C.

According to the distribution model, the observed forgetting curve should depend on what slice of the distribution is being captured in the data. If overall performance is high—for example, if percent correct ranges from 50% to 95%—the distribution model predicts that compared to the item forgetting rate, the observed forgetting rate should become flatter and closer to linear (or even progress beyond linear and switch from being convex to concave, as we explain later). The reasoning behind this prediction can be seen in Figure 2 and Figure 3. In the left half of Figure 2, the shape of the distribution drives the curve to be concave (like the right side of a frown). At the same time, as shown in Figure 3, being forgotten along a power function drives the curve to be convex (like the left half of a smile). These concave and convex forces tend to neutralize each other, leading to a curve that tends toward linearity, as shown in Figure 4, in the area to the left of the dashed vertical line (note: Figure 4 is an enlarged version of Figure 3C). In short, at high rates of recall, the effect of the distribution is to make the observed forgetting curve closer to linear than the underlying item forgetting curve.

If overall performance levels are low (e.g., below 50%), however, the radius of the forgetting curve should decrease (i.e., the curve should become curvier). When the recall performance is low, the distribution drives the curve to be like the left side of a smile (see the right half of Figure 2), and so does the power function. Therefore, the distribution amplifies the effect of the item forgetting rate’s power function, as shown in Figure 4 in the area to the right of the dashed line. Thus, at low levels of performance, the observed forgetting curve is more curved (i.e., has a smaller radius) than the item forgetting curve.

Empirical support for these predictions comes from an article called “Linear Forgetting” by Fisher and Radvansky (2019). They conducted three studies and reviewed many additional past studies, all showing linear forgetting. They conclude that “an increased degree of learning appears to be important for linear forgetting to occur” (p. 18). This finding is what the distribution model would predict. (Note that Fisher and Radvansky say, “higher degrees of learning and meaningfully complex materials may be jointly needed to observe linear forgetting.” Predictions about complexity, although important, are beyond the scope of this article. Also, note that other studies have not found a relationship between the rate of forgetting and the degree of initial learning; see Rivera-Lares et al., 2022, 2023).

### 3.2. Predictions Based on the Shape of the Distribution

The distribution model predicts that, all else being equal, the shape of a distribution should affect the empirically observed forgetting curve. This prediction was tested by Kornell et al. (2011) in a study that examined forgetting rates when underlying distributions had different shapes. Their research compared two conditions. In one condition, participants studied a set of items and presumably developed a roughly normal (or at least unimodal) distribution of memory strengths. In the other condition, participants were tested without feedback. In this case, the items that were recalled correctly, which were already above the recall threshold by virtue of being recalled, moved farther above the threshold because of retrieval practice effects (e.g., Rowland, 2014). The weaker items, which were not retrieved and thus were already below the threshold, were not strengthened because following retrieval failure, the correct answer was not revealed. This second condition, therefore, produced a bifurcated distribution with some items well above the recall threshold and some below it.

The distribution model predicts that a bifurcated distribution like this should appear to be forgotten relatively slowly compared to a unimodal distribution. Forgetting happens when items cross the threshold, and in these bifurcated distributions, the items that started out above the threshold were strong enough that they were well above the threshold. It should take longer for such items to start crossing the threshold, which translates to slower forgetting. By contrast, the other condition, with its normal distribution, should be forgotten more quickly because there were items just slightly above the threshold, and these items should cross the threshold relatively quickly. This prediction of the distribution model was supported by the data.

Furthermore, in Kornell et al.’s (2011) research, there was an additional condition in which participants were tested and given feedback. This condition should not result in a bifurcated distribution because all items could be learned (i.e., potentially move above the threshold) via feedback even if they were not recalled during the study phase (Retrieval attempts followed by feedback enhance learning to roughly the same degree as successful retrieval attempts. See Grimaldi & Karpicke, 2012; Knight et al., 2012; Kornell et al., 2009). In this condition, the distribution model predicts that the observed amount of forgetting should be similar in the testing and study-only conditions, because in both cases the underlying item distribution is expected to be unimodal and roughly normal. This prediction was also supported (Note that the tested items should be farther above the threshold than the studied items, which should affect the observed forgetting rate as shown in Figure 2; however, because there was not a large difference in the mean accuracy scores and thus the positions of the distributions, this effect should be relatively small). In short, these findings showed that the shape of item distributions impacted the rate at which items appeared to be forgotten as predicted by the distribution model.

On this topic, it should be noted that the distribution model is not contingent on the shape of the distribution being normal, or any other shape. The distribution of item strengths should impact the observed forgetting rate as long as different areas of the distribution contain different numbers of items (e.g., there are peaks and low points in the distribution). This claim is related to the topic we discuss next.

### 3.3. Predictions Based on the Standard Deviation of Distribution

The distribution model predicts that item distributions will have a larger effect on percent correct when the standard deviation of the distribution is smaller. As an extreme example, if the standard deviation was very small and all of the items had roughly the same strength, then they could all pass the threshold at basically the same time. In this case, the empirically observed forgetting curve would look like a step function; it would sit at 100% correct until the item distribution passed the threshold, at which point it would plummet to 0% correct. This would be predicted even if the underlying item forgetting rate was a power function (or any other function).

At the other extreme, when the standard deviation is large, the distribution would behave similarly to uniform, or rectangular, distribution. In other words, there would be about the same number of items at any given point in the distribution. In this case, the distribution’s effect would be minimal, and the observed forgetting curve would look roughly the same as the underlying item forgetting curve. In short, a distribution with a smaller standard deviation should have a larger effect on the observed forgetting curve.

There is no strong evidence in support of these predictions. However, indirect support comes from the studies reviewed by Fisher and Radvansky (2019). A common theme among studies that showed linear forgetting was that participants learned to a criterion (i.e., stopped studying after answering correctly a set number of times). Learning to criterion should decrease the standard deviation of item strengths. To illustrate the effect of learning to criterion, assume participants study a set of items to a criterion of three correct responses. When they get an item correct for the third time, it has reached a certain level of strength. At this point, it is no longer studied. As a result, all items reach a similar level of strength. Thus, learning to criterion should decrease the standard deviation of a distribution of memory strengths by ensuring a similar level of learning for all items. One reason this evidence does not provide strong support for the prediction, however, is that learning to criterion simultaneously moves items above the threshold and it decreases their standard deviation. Because these two changes are confounded, it is difficult to know which one impacts empirical forgetting curves. Stronger conclusions could be drawn if future research can unconfound these variables (for example, create a low standard deviation without moving items far above the threshold).

In summary, learning to criterion should make the standard deviation relatively small, which should cause the shape of the distribution to have a relatively larger impact on the observed forgetting curve. Thus, the distribution model predicts that, all else being equal, learning to criterion paradigms should be especially likely to produce linear forgetting for two reasons: the shape of the distribution should have a pronounced effect and, because learning to criterion produces relatively high levels of initial recall, this effect should flatten the distribution (see the portion of Figure 4 to the left of the dashed line). This prediction appears to be consistent with the data reviewed by Fisher and Radvansky (2019). Next, we describe a study that further tested this prediction.

## 4. Experimental Investigation

We conducted an experiment to test a prediction of the distribution model: The typical convex forgetting curve can go beyond linear and become concave. That is, instead of looking like the left side of a smile, or even a straight line, it might look like the right side of a frown. This prediction does not emerge naturally from models that assume forgetting follows a power or exponential function. Thus, if the data fit a concave curve, it would support the distribution model.

In designing the study, we attempted to create conditions that would take advantage of two predictions we described earlier. (1) In a distribution with a high level of memory strength, the effect of the distribution should be to drive the percent-correct curve toward being a frown shape. (2) When a distribution of items has a small standard deviation, it should amplify the effect of the distribution. We created a high level of memory strength and a small standard deviation by only testing participants on items they had answered correctly during the study phase. The retention interval between studying and testing served as the independent variable.

### 4.1. Method

Participants studied a set of word pairs, took an initial test, and then took a final test. The retention interval between the initial and final test was 2, 20, or 80 other items. The study’s method and data analysis plan were pre-registered at https://osf.io/fpx6d/ (accessed on 21 May 2025).

### 4.2. Participants

The final sample consisted of 99 participants. Additional participants were removed from the analyses, in accordance with the pre-registration, because they indicated that they had done the experiment before (four participants) or because they left more than half of the answers blank on the final test (two participants). Fourteen additional participants answered zero questions correctly during restudy in at least one condition; because we only analyzed items that were answered correctly during restudy, these participants had no data for at least one condition and were excluded from the analyses.

Participants were paid $2.00. They were recruited using Amazon Mechanical Turk during late January and early February of 2020. Their average age was 38 (*SD* = 10.9, range = 23–71); 53 reported being female, 45 reported being male, and one did not report their gender. All participants reported that they lived in the United States.

### 4.3. Materials

The materials were 55 unrelated word pairs such as “sunset-pudding.” The words were taken from Paivio et al. (1968). Words were selected if they had an imagery score of 6 or 7 (on a 1–7 scale), a concreteness score of 6 or 7 (on a 1–7 scale), and contained between four and twelve letters. After 110 words were selected, they were paired randomly.

### 4.4. Procedure

The study was conducted online. Participants encountered each word pair three times during the experiment. The first time was a copy trial. The full pair was shown (e.g., trumpet-house) and below it, the cue word was shown again (e.g., trumpet) and participants were asked to type in the target (e.g., house).

After six intervening trials, participants encountered the pair again. This time, they were given a test trial: They were shown the cue word and asked to type in the target. They were not shown the target after making their response.

The third encounter with a given word pair was another test, again with no feedback. Between the second and third trial, there was a retention interval of 2, 20, or 80 other trials.

A total of 30 pairs were included in the data analysis, with ten pairs in each retention interval. To assign pairs to conditions, a unique randomization was used for each participant.

An additional 25 pairs were shown during the study. Four of these pairs were used during an initial practice phase that consisted of four copy trials followed by four test trials on the same items. Another 21 pairs were used in filler trials that had to be mixed into the other trials in order to achieve the desired retention intervals.

## 5. Results

In accordance with the pre-registration, items were only analyzed if participants answered them correctly on the initial test. Thirty-three percent of items were excluded. This exclusion alone should not produce concave forgetting, unless the distribution model’s predictions are supported. It also had two desired effects: (1) It ensured that all items were above the threshold at the start of the retention interval, and (2) it decreased the standard deviation of item strengths by excluding the weaker items, leaving only a smaller, more tightly clustered set of stronger items.

**Figure 5:**
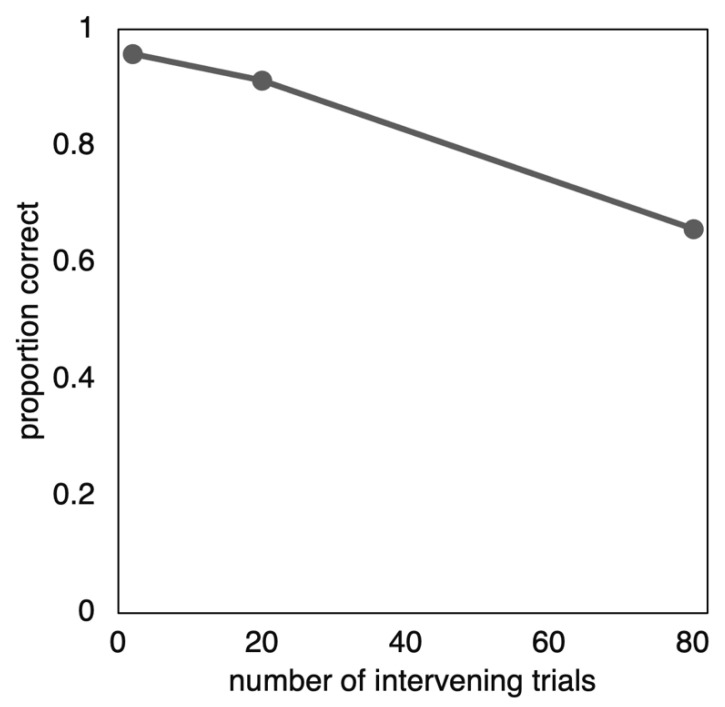
Proportion correct curve showing a concave pattern. Most forgetting curves are convex.

We predicted that the rate of forgetting would be concave; that is, it would become steeper with time. This prediction was supported; the rate of forgetting (i.e., the slope of the line in Figure 5) was steeper for the second interval, between time 2 and 3, than it was for the first interval, between time 1 and 2. On the final test, the proportion correct was *M* = 0.958, *SD* = 0.110 for time 1; *M* = 0.913, *SD* = 0.158 for time 2; and *M* = 0.658, *SD* = 0.298 for time 3.

To test whether the increase in slope was significant, we computed forgetting per unit of time for interval 1 and interval 2 for each participant. Forgetting per unit of time is equivalent to the slope of the line. For example, if a participant’s percent correct scores were 95, 90, and 70 percent, then the amount forgotten for the two intervals would be 95 − 90 = 5 items and 90 − 70 = 20 items, but the two forgetting per unit of time scores would be (95 − 90)/(20 − 2) = 0.28 and (90 − 65)/(80 − 20) = 0.42. In these equations, the denominator is the number of items separating the two tests; for example, the first and second retention intervals were 2 and 20 items, so we divided by the difference, which is 18 items (To avoid confusion, we should point out that participants were not tested on the same item twice).

The forgetting per unit of time was closer to flat for the first interval (*M* = −0.254, *SD* = 0.877) than the second interval (*M* = −0.424, *SD* = 0.481). When comparing these slopes, a Shapiro–Wilk normality test showed a significant violation of normality (W = 0.0913, *p* < 0.001). Therefore, instead of a paired *t*-test, we compared the scores using a Wilcoxon W test. The difference was significant: *W* = 1257, *p* = 0.019, and rank biserial correlation = 0.296.

## 6. Discussion

This study showed that in terms of percent correct, forgetting was slower at first and then increased in the second interval. This finding contrasts with most previous research in which forgetting is relatively quick at first and appears to slow down (e.g., Ebbinghaus, 1885; Loftus, 1985).

The distribution model predicts this finding for the reasons illustrated in Figure 2, Figure 3 and Figure 4. It seems safe to assume that individual items were forgotten in accordance with something like a power or exponential function (i.e., quickly at first and then more slowly). Because of the high rate of initial recall and (we assume) small standard deviation of item strengths, the distribution model predicts that the effect of the power or exponential function was weaker than the opposing effect of the distribution. This finding is a more extreme version of the apparent linear forgetting that has been found in other studies (Fisher & Radvansky, 2019); in this case, the distribution caused the curve to go past linear and become concave.

It is worth noting that the retention intervals in this study were relatively short compared to other forgetting research (e.g., Wickens, 1998; Wixted, 2004). Whether these findings would generalize to longer time scales is a question for future research. Relatedly, the short retention intervals led to high levels of performance. Thus, we cannot rule out the possibility that ceiling effects played a role in these findings. However, it is not clear that ceiling effects would produce a concave forgetting curve, and moreover, very high levels of performance may be necessary to observe concave forgetting, according to the distribution model.

## 7. A More Complex Distribution Model

Thus far, we have considered a relatively simple distribution model with two key assumptions: Items have a distribution of different memory strengths, and there is a recall threshold. A more complex model could make additional assumptions; for example, the position of items might not be the only thing that changes; if threshold positions were to change, it would also impact the shape of the empirical forgetting curve. Furthermore, item retrievability might momentarily fluctuate, and upward fluctuation could depend on what retrieval cues were present at the time of retrieval. Upward fluctuations in item strength or downward movement of the thresholds could both help explain the finding, from the reminiscence and hypermnesia literatures, that retrieval success can increase over time (Payne, 1987; Roediger & Payne, 1982).

We will examine what happens when a different assumption is added to the model, that different items have different forgetting rates. That is, two memories could differ in terms of both their current level of strength and the rate at which they lose strength (i.e., are forgotten). Our simple model assumed that each memory had a unique level of strength, but to capture both current strength and forgetting rate, a more complex model would need to assume that memories are characterized by more than one strength variable.

The New Theory of Disuse (Bjork & Bjork, 1992, 2006) assumes that a given memory is characterized by two kinds of strength. Retrieval strength is how accessible a memory is at the moment of retrieval, while storage strength is how strongly the memory is stored on a long-term basis. Retrieval strength is the only kind of strength that can be measured directly, and therefore it is the type of strength we have discussed thus far—it determines whether a memory is retrieved at any given time. Storage strength, by contrast, controls the rate at which the memory is forgotten; memories with more storage strength are forgotten at lower rates. Thus, the New Theory of Disuse provides a theoretical foundation for assuming current strength and forgetting rate are separate attributes of a memory.

**Figure 6:**
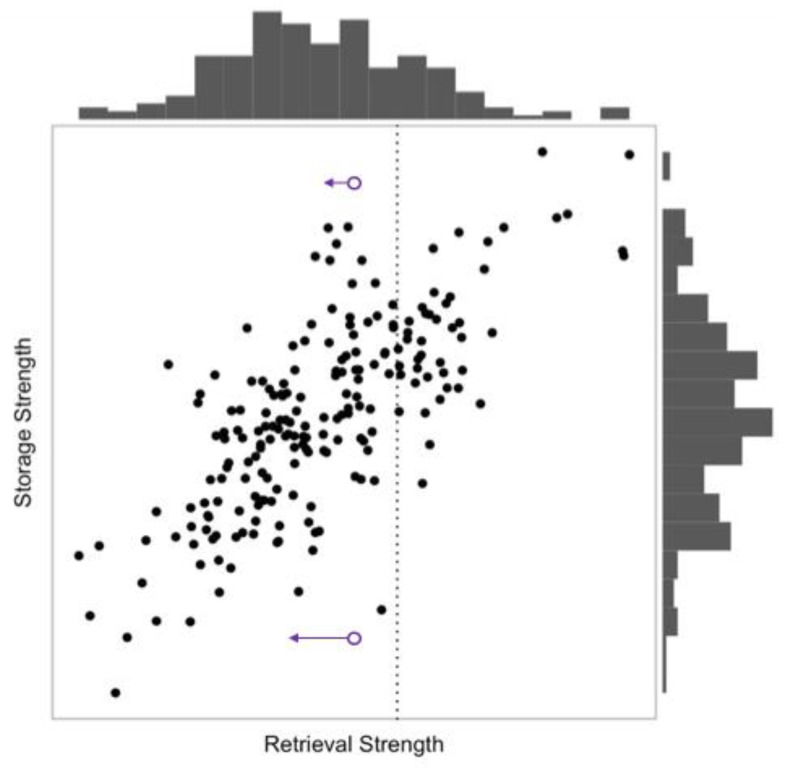
A hypothetical scatter plot with histograms in the margins. Each point in the scatter plot represents a memory. Retrieval strength is on the horizontal axis. The histogram above the scatter plot is the distribution of retrieval strengths based on the values in the scatter plot, which is comparable to the distribution of memory strengths in previous figures (e.g., Figure 1). The dashed vertical line represents the retrieval threshold. Storage strength is on the vertical axis, and it is represented by the histogram to the right of the scatter plot. Items with more storage strength tend to have more retrieval strength, creating a positive correlation. The two unfilled purple circles represent two items with the same initial retrieval strength. The lines attached to the circles represent the amount of forgetting in a set amount of time. The different line lengths show that items with less storage strength are forgotten (i.e., lose retrieval strength) more quickly than items with more storage strength.

A more complex version of the distribution model could assume that different items have different initial levels of strength and different forgetting rates—that is, that a given memory could be represented in two different distributions: a retrievability distribution and a separate forgetting rate distribution. Figure 6 illustrates such a bivariate distribution.

Introducing variable forgetting rates into the model has the potential to make it significantly more complex. From a mathematical perspective, initial strength is a scalar value. It is easy to compute averages of scalar values. Forgetting rates, however, are represented by functions. If each item has its own forgetting function, then averaging across items becomes complicated because averaging across multiple functions does not necessarily return the same function. For example, when multiple exponential forgetting rates are averaged, the result is often fit best by a power function (R. B. Anderson, 2001; R. B. Anderson & Tweney, 1997; Fisher & Radvansky, 2019; Murre & Chessa, 2011). This finding implies that when an empirical forgetting curve appears to be a power function, the underlying forgetting rates of the individual items might not be a power function.

Next, we describe a distribution model that includes variable forgetting rates. Such a model would depend on the nature of the forgetting function. The following equation is an exponential function:

f(t) = s − a^t^

The variable t represents time, a is a constant that represents the item’s forgetting rate, and s is the item’s initial strength. In the simple distribution model described earlier, any given memory had its own unique value of s. In a more complex version of the model, each item would also have a unique value for a. Thus, instead of there being one distribution, there would be two, for s and a (see Figure 6).

A power function is similar to an exponential, but it has the following form:

f(t) = s − a × t^b^

A complex model could posit that there are three unique values for each item: s, a, and b. Thus, there could be three distributions, for s, a, and b.

If items are forgotten at different rates, then there is an additional complication: Over time there will be changes in the shape of the distribution of item retrieval strengths. Thus far, we have assumed that the mean, standard deviation, and shape of the distribution remain the same as they move leftward over time. However, differing forgetting rates should cause the shape of a distribution to morph as time passes. The nature of this morphing process depends upon the items’ forgetting functions. It also depends on the correlation between an item’s retrieval strength and its forgetting function; as Figure 6 shows, there should be a correlation between retrieval strength and storage strength—that is, between and item’s current retrieval strength and its rate of forgetting—such that an item that is higher in retrieval strength will also tend to be forgotten more slowly. Because of this correlation, items with high retrieval strength (on the right side of Figure 6) also tend to have relatively high storage strengths, and thus they will move leftward (i.e., be forgotten) at relatively low rates; by contrast, items with lower retrieval strength will lose retrieval strength relatively quickly (i.e., items on the left side of Figure 6 will move leftward relatively quickly).

This morphing process has two implications. First, the distribution of retrieval strengths should grow wider and more negatively skewed over time. Second, above we made the assumption that the underlying distribution of memory strengths was bell-shaped (i.e., a normal distribution). This assumption was useful for illustration purposes, but the reality is that we do not know how memory strengths are initially distributed. Because of the morphing process, how they are distributed after a delay has no singular answer but rather changes over time.

We created a simple Monte Carlo model to simulate forgetting in a complex version of the distribution model. The key parameters of the model, which seem faithful to the underlying cognitive processes, were a mean and standard deviation for initial memory strength and a mean and standard deviation for each parameter in the forgetting functions. It was easy to closely fit empirical data using this model by playing around with parameter values. Power functions and exponential functions could fit the same dataset equally well, and the data could be fit using different assumptions about the shape of the initial memory strength distribution (e.g., normal or rectangular). We mention this model for two main reasons. The first is to illustrate the key parameters. The second is to point out a weakness: When a model has so many free parameters, its ability to fit data is not good evidence that it is an accurate model (Roberts & Pashler, 2000). Furthermore, it is difficult to test such a model because it can be used to predict so many patterns of data. Perhaps future refinements of this kind of modelling will be able to address these issues.

## 8. The Importance of the Threshold

It is worth noting that the threshold is a crucial aspect of the distribution model because all the predictions of the model stem from the position of items relative to the threshold. The idea of a threshold goes hand in hand with the fact that correct answers are either recalled or not recalled (i.e., above or below the threshold).

When responses are either correct or incorrect, it becomes impossible to directly observe an item’s forgetting rate—one can only observe whether it is currently above or below the threshold. If memory strength could be measured in a way that did not involve a threshold, it might be possible to observe individual forgetting rates unaffected by the shape or position of the distribution. One possible measure is response time, which is a continuum without a threshold. Response time is typically analyzed only after correct responses, which neutralizes the effect of the threshold because all responses would be for items that were above the threshold. Future research could use response time-based paradigms to shed light on item forgetting rates.

## 9. Educational Implications

Forgetting is an important aspect of education because there is little point in learning if the memories do not stick (P. C. Brown et al., 2014). The distribution model of forgetting has implications for education. Students often prepare for exams by studying a fact or concept enough to get the answer right on the exam, but not more. Doing this is similar to learning to criterion, where the criterion is the minimum amount of study required for good performance. Essentially, they reach a certain level of confidence and, to save time, decide “I can stop studying this one.” This strategy is understandable and extremely common.

The distribution model suggests that this strategy has an important downside: Studying just enough to ace an exam will save time and produce good grades, but it will also maximize the loss of knowledge due to forgetting. To understand why, consider a student who studies for a quiz with 20 questions. Studying just enough means pushing those 20 items just slightly above their recall threshold, but not further. When a memory is near a threshold, a short period of time is enough for forgetting to drop it below the threshold. Thus, the distribution model can help explain why students who do well on exams frequently find that they do not remember what they learned for very long: Regardless of the shape of the forgetting curve, items close to the threshold will soon be forgotten.

Similarly, for educators, the distribution model helps explain an all-too-common outcome: When students do well on an exam, it is not safe to assume they have achieved long-term learning. Studying beyond a minimum threshold takes effort, but it also creates long-lasting memories, which is a core goal of education.

## 10. Concluding Comment

We have argued that distributions play a crucial, though often overlooked, role in the shape of empirical forgetting curves. In future research, considering and accounting for the impact of distributions might be a step toward understanding the underlying item forgetting rates, which are arguably the key cognitive process in forgetting.

Considering distributions might also be useful in other areas of psychology research. For example, distributions of memory strength relative to thresholds are relevant when learning causes memory strength to increase. In psychology more generally, if thresholds are relevant in an area of research, then it may be worthwhile to consider the movement of distributions relative to the thresholds.

## Data Availability

The original contributions presented in the study are included in the article; further inquiries can be directed to the corresponding author.
